# Exploring potential environmental drivers of migration phenology in two Mongolian Plateau-nesting goose species

**DOI:** 10.1186/s40462-025-00583-0

**Published:** 2025-08-14

**Authors:** Junjian Zhang, Xianghuang Li, Xueqin Deng, Iderbat Damba, Nyambayar Batbayar, Zhenggang Xu, Yong Zhang, Lei Cao, Anthony David Fox

**Affiliations:** 1 State Key Laboratory of Urban and Regional Ecology, Research Center for Eco-Environmental Sciences, Chinese Academy of Sciences, Beijing, China; 2 East China Sea Survey Center, Ministry of Natural Resources, Shanghai, China; 3 Center for Environmental Education and Communications (CEEC) of Ministry of Ecology and Environment, Beijing, China; 4 Institute of Biology, Mongolian Academy of Sciences, Ulaanbaatar, Mongolia; 5 Wildlife Science and Conservation Center, B-1301 Union Building, Sukhbaatar District, Ulaanbaatar, Mongolia; 6 Hunan Engineering Research Center of Ecological Environment Intelligent Monitoring and Disaster Prevention and Mitigation Technology in Dongting Lake Region, College of Information and Electronic Engineering, Hunan City University, Yiyang, China; 7 Co-Innovation Center for Sustainable Forestry in Southern China, College of Life Sciences, Nanjing Forestry University, Nanjing, China; 8 University of Chinese Academy of Sciences, Beijing, China; 9 Department of Ecoscience, Aarhus University, Aarhus, Denmark

**Keywords:** *Anser anser*, *Anser cygnoides*, Migration phenology, NDVI, Snow cover, Temperature, Vegetation growth

## Abstract

**Background:**

Migratory phenology affects fitness and therefore plays a crucial role in the annual life cycle of migrants. Various indicators in relation to the migration patterns of Arctic nesting birds have been well studied (e.g. vegetation production), but we still lack knowledge from lower latitudes, e.g. the Mongolian Plateau, which is one of the top-priority regions for avian research and conservation.

**Methods:**

We used 208 spring and 248 autumn migration tracks from individually tagged Swan Geese *Anser cygnoides* (SG) and Greylag Geese *A. anser* (GG) from four geographically discrete breeding groups across the Mongolian Plateau. We analyzed the difference in their migratory timing, how they responded to nine environmental metrics as indicators of environmental change, and the probability of spring arrival and autumn departure.

**Results:**

We found significant differences in spring and autumn departure times between species, yet their arrival times were similar, although the migration phenology of eastern nesting birds differed significantly from those in central and western Mongolia. Their spring migration followed the onset of daily temperature reaching 0 °C, but was not correlated with indices of plant green-up, which occurred behind them along their migration routes. The autumn departure phenology of SG exhibited stronger responses to 0 °C nighttime temperatures, while the GG responded more to 0 °C cumulative temperatures.

**Conclusions:**

Two goose species follow behind the daily 0 °C and before the green-up of plants in spring, allowing the time of hatching of goslings to coincide with the plant growth peak, ensuring a predictable food supply for the nidifugous juveniles. Vegetation and snow metrics were not appropriate indicators to predict the migration process of either species, due to the lack of strong latitudinal gradients in plant growth and long-term snow cover.

**Supplementary Information:**

The online version contains supplementary material available at 10.1186/s40462-025-00583-0.

## Background

The timing of key events in the annual cycle of migratory animals can have crucial effects on their fitness [1]. Due mainly to macro-, but also micro-environmental factors, migrants’ local food availability often fluctuates cyclically between abundance and scarcity, influencing migrants’ decision-making about when precisely to undertake critical elements of their migration between breeding, staging and survival habitats [*sensu*^2^–^4^]. Although the timing is partly internally controlled [5], migratory birds can quickly adjust to various external environmental cues during migration [6]. Under a warming climate, birds are constantly facing the need to adjust their migration patterns to maximize fitness [7], but this is especially important for migrants towards the more capital-breeding end of the capital/income breeder spectrum, which subsidize investment in reproduction from stores accumulated prior to breeding [8]. For such forms, individuals need to aim for efficient fueling (storing both energy and nutrients) to invest in ultimately successful breeding. However, they may still suffer from a phenological mismatch if climate change has shifted the timing of food abundance for offspring relative to the timing of optimal egg-laying [9]. To effectively understand the ability of specific taxa to respond to such changes and to develop appropriate conservation mitigation strategies, we need to know which factors most influence the timing of critical stages of bird migration throughout the annual cycle [10].

Various hypotheses concerning the environmental factors that influence birds’ migration have been proposed. For instance, the green wave hypothesis suggests that herbivores (such as northern nesting geese) time their spring migration to coincide with the onset of plant growth and nutrient peaks along their migratory corridor to the breeding grounds [11–13]. In the final leg of their migration, geese attempt to reach their breeding grounds as early as possible, before the snow melts, to occupy favorable territories and increase their breeding success [14–17]. In contrast, the late season cessation of vegetation growth and increasingly poor weather are significant triggers for individuals to embark on autumn migration [18]. At this stage, declines in vegetation greenness impact invertebrate activity which can indicate the start and end of the autumn migration peaks of insectivorous birds [19]. Moreover, the arrival of frost serves as a cue for the autumn migration of large-bodied waterbirds away from breeding and stopover sites [20, 21]. However, support for these hypotheses has mostly been sought among Arctic-breeding species [17, 20, 22–24] so we lack knowledge from further south, as for example on the Mongolian Plateau, known to be of importance for several key regional waterbird populations [25].

The arid continental grasslands of the Mongolian Plateau are experiencing higher warming trends than the global average because of their high latitude and elevation [26]. They are situated at the junction of three different waterbird migratory flyways, and provide globally significant breeding grounds for several threatened species [27]. Even the critically endangered Siberian Crane *Leucogeranus leucogeranus*, which has traditionally bred in the Arctic, has begun a tradition among sub-adults to summer here, potentially to mitigate recent adverse effects of extreme climatic events on the summering range [28]. In addition to climate change, the Mongolian Plateau is increasingly affected by anthropogenic disruption (i.e. grassland degradation caused by overgrazing) [29], and since the 1980s has entered a cyclical period of severe drought, resulting in significant declines in number of highland lakes in this region, which will persist in the coming decades [30]. Because these wetlands are crucial as breeding sites and migratory stopovers for migratory birds [31], the Mongolian Plateau is one of the top-priority regions for avian research and conservation in the light of the predicted effects of global environmental change there [32].

Large-bodied migratory birds (including geese) migrate by flapping flight, making them particularly susceptible to environmental changes due to a variety of physiological constraints and higher energy requirements for flight [33–36]. Three goose species breed on the Mongolian Plateau: Bar-headed Goose *Anser indicus*, Swan Goose *A. cygnoides* and Greylag Goose *A. anser* [37]. Bar-headed Geese migrate within the Central Asian flyway and currently show increasing numbers [38], while Swan Goose and Greylag Goose (abbreviated as SG and GG) belong to the East Asian-Australian flyway and have exhibited different population trends over the past 20 years, with the SG decreasing from 78,000 to 54,000 and the GG increasing from 3,300 to 30,000 [39, 40]. While the environmental drivers of Arctic breeding goose migration phenology have been relatively well-studied, those of SG [20, 22, 41] and GG [42–46] are less well understood and are unknown in East Asia. For these reasons, these two related species with similar breeding and wintering areas but differing population trends, offer an invaluable basis for migratory phenology comparative studies.

In this study, we used telemetry data from individually tagged SG and GG from four breeding groups distributed across the Mongolian Plateau from 2015 to 2023. We aimed to (1) quantify and compare the timing of spring and autumn migrations within and between species; (2) test whether geese migrate in response to environmental factors, such as temperature, vegetation growth, and snow cover; (3) determine the association between spring arrival and autumn departure probability of geese and these environmental factors. Our ultimate aim is to deepen our understanding of the factors affecting the timing of bird migration, to provide a better basis for conservation management to mitigate, where possible, the adverse effects of land-use and climate change, through the comparative analyses of their migratory phenology and their relationships to environmental factors.

## Methods

### Animal capture and transmitter deployment

**Fig. 1:**
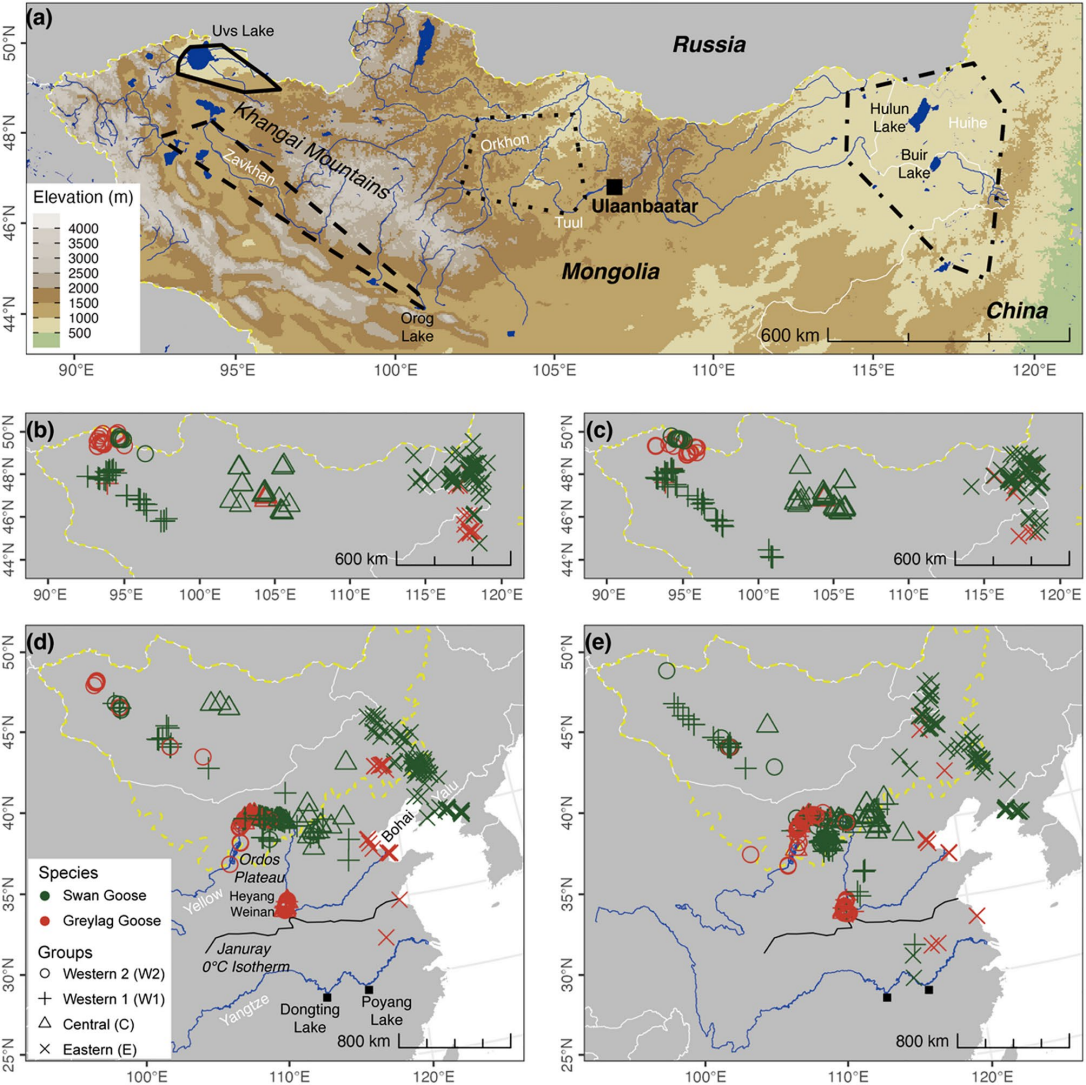
The migration nodes of tracked Swan Goose (*Anser cygnoides*) and Greylag Goose (*A. anser*). **a**, summering grounds of four groups bred across the Mongolian Plateau, drawn from the Minimum Convex Polygon home range; **b** & **c**, ended points of spring migration and started points of autumn migration respectively; **d** & **e**, stopover sites in two seasons. The yellow dashed lines are the boundary of Mongolian Plateau, defined as the region consisting of Mongolia and the Inner Mongolia Autonomous Region of China; the white lines, boundary of countries; the blue lines, rivers; the black lines, the average zero-degree isotherm line in January

The SG and GG breed across the Mongolian Plateau [39, 40] and mainly winter in the middle and lower Yangtze River floodplain [47, 48]. The distributions of both species can be divided into four groups based on differences in the elevation and grassland types (Fig. 1a). (1) Western 1 Group (hereafter W1): centered on wetlands around Khyargas, Khar-Us and Khar Lakes, and along the Zavkhan River (92–101°E, 45–49°N), in semi-desert grassland [49] at an elevation of around 1300 m. (2) Western 2 Group (W2) with core breeding areas in wetlands around Uvs Lake (92–96°E, 49.5–50.8°N), in semi-desert grassland at elevations around 800 m, separated from W1 by the Khangai Mountains. (3) Central Group (C) distributed in wetlands along the Orkhon and Tuul Rivers (102–106°E, 47–50°N), in temperate grassland around 950 m above sea level. (4) Eastern Group (E): wetlands in the Dauria Region, along the Huihe River, and Xilinhot Grassland (115–120°E, 45–50°N), in the temperate grassland region at elevations around 700 m. The groups were located *c.* 1,000 km apart from each other, except for the two western groups (*c.* 200 km), so are geographically discrete.

A total of 244 Swan Geese and 68 Greylag Geese were captured during flightless moult from 2017 to 2019. The capture locations included Uvs Lake (50.4° N, 93.1° E), Bayan Lake in Zuungobi (49.9° N, 93.9° E), Khar-Us Lake (47.8° N, 92.2° E), Dorgon Lake (47.6° N, 93.4° E) and Taigan Lake (46.4° N, 97.4° E) in western Mongolia; Ugii Lake (47.8° N, 102.7° E) and Bayan Lake in Dashinchilen (47.9° N, 104.3° E) in central Mongolia; and Dauria International Protected Area (47.7° N, 117.5° E) in eastern Mongolia. In addition, 12 Greylag Geese were also captured at Poyang Lake (29.0° N, 116.4° E) and the Anhui Lakes (30.9° N, 117.7° E) on the wintering grounds in China during the winters of 2014/15 and 2016/17 which migrated back to these areas and are also considered here.

Geese were fitted with solar-charged telemetry devices integrated into neckbands produced by different manufacturers. We deployed 251 Druidtech (China, 35 g, 2017 and 2019) and 61 Ornitela (Lithuania, 44 g, 2018 and 2019) devices for geese captured in summer, and eight Druidtech (China, 35–45 g, 2016) and four Global Messenger (China, 26 g, 2014) in winter. All these devices constituted less than 3% of the geese’s body mass (with an average of 1.3%) to minimize the impact on their behaviour and ecology [50]. The devices provided date/time stamped GPS/Beidou positions with a horizontal accuracy of within 10 m, and the data was uploaded via GSM networks. Issues such as low battery power, signal loss, and device failure affected the accumulation of regular and precise data, especially during the winter when daylight was short, and the solar angle was low. As a result, only migration tracks with 2–288 fixes per day were used for the study, resulting in 208 spring and 248 autumn migration records with sufficient precision for analysis (Table 1). These records included 156 spring (2018–2023) and 187 autumn (2017–2023) tracks for SG, 52 spring (2015–2023) and 61 autumn (2015–2022) for GG. Due to signal loss, two spring and seven autumn migrations of SG and two autumn migrations of GG were not completed.

### Definition of migration parameters

We defined spring migration as starting from the first position after tracked birds departed from the wintering areas and ending with the first position received from the breeding grounds [51]. Likewise, we defined autumn migration based on similar criteria for the return to the wintering areas. Non-breeding and failed breeding geese undertook long-distance moult migrations during the summer (c. 200 km, see Fig. S1) and accumulated sufficient energy reserves before autumn migration [52], so for these birds autumn migration initiation was defined from moulting areas, while for breeders, this was from the breeding sites. To segment movement tracks and identify stopover sites, we followed the methods described by Wang et al.(2018; [53]). Departure date was defined as the date of the first position when the individual left its wintering/breeding/moulting areas, determined by the method above to have acquired flight status. Arrival date was defined as the date of the first time when the individual was judged to have reached its final destination, based on the methods above to qualify as non-flight status after a period of flight. The overall distributions of individual migration timings from all individuals can be seen in Fig. S2. A site where a bird rests and feeds for over two days during migration is defined as a stopover site [12, 54]. Consequently, we excluded those sites where birds likely only rested briefly or drank [55].

### Environmental variables

Nine environmental metrics used as proxies for environmental cues potentially influencing the timing of migration events were analyzed as follows: average and nighttime Land Surface Temperature indexes (LSTa and LSTn), average and cumulative air temperature indexes (Ta and Tcum), two plant growing indexes (start of season, SOS, and Greenup for spring; end of season, EOS, and Dormancy for autumn), and a snow cover index.

We utilized the MODIS 1KM Land Surface Temperature 8-Day products MOD11A2 from 2015 to 2023 to extract LST data for analysis [56]. LSTa and LSTn were defined as the first day when the daily average and nighttime land surface temperature exceeded (during spring) and fell below (during fall) 0 °C. When the LSTa exceeds 0 °C, the surface snow begins to melt, initiating grass green-up; conversely, when the LSTn falls below 0 °C, it presages frozen soils and surface water which results in shortages of food and water resources [20, 57]. Xu and Si(2019; [20]) referred to LSTn as “frost day” and considered it to be a primary cue in autumn departure decisions for geese in Far Eastern Asia. To ensure reliable results, we only retained pixels with errors below 2 °C, based on the quality indicators files.

Air temperature data were obtained from the Global Land Data Assimilation System (GLDAS) on a 0.25° × 0.25° fixed grid at a daily temporal resolution from 2015 to 2022. Ta and Tcum were computed as the first day when daily and 2-week cumulative air temperatures exceed and fall below 0℃ in two seasons. Two indexes have previously been confirmed to show significant relationships with the autumn departure of cranes and geese [20, 21]. We excluded sites where Ta and Tcum in spring appeared before the 30th day of the year, which occurs near the zero-degree isotherm in January.

Normalized Difference Vegetation Index (NDVI) is commonly used as a basis to describe vegetation growth characteristics [58]. We utilized the MODIS 1KM Vegetation Indices 16-Day products MOD13A2 from 2015 to 2023 to extract vegetation phenology for analysis [59]. For each year, we applied a time series model to the raw NDVI values for each pixel. Initially, we used weighted Whittaker (*wWHIT*) for a rough fitting, which is optimal for vegetation types that undergo rapid seasonal changes during the growing season [60]. Following this, we used the Asymmetric Gaussian (AG) fit to capture rapid changes in vegetation during every growing season [61]. Finally, we employed the Threshold and Inflection methods to calculate the start and end of the growing season in spring and autumn respectively (Fig. S3). SOS and EOS were the dates when the curve of the NDVI annual amplitude within the year reached 50% in the two seasons (i.e. the days with peak green-up and dormancy rate). Greenup was the first local maxima and Dormancy was the last local minima of the NDVI rate of change [62]. Wang et al.(2019; [22]) pointed out SOS could explain observed migration patterns of a few grazing populations, while it cannot be considered a ubiquitous driver of herbivorous waterfowl spring migration. We excluded pixels with anomalous spring phenology where the SOS and Greenup index occurred (1) before the 30th day of the year, or (2) after the 240th day of the year, or (3) later than the time when the EOS and Dormancy index occurs, which often signifies essentially non-vegetated areas [22]. Furthermore, we discarded pixels with very low NDVI values (*c.* 19% of pixels with the annual mean NDVI < 0.1) [63]. All plant growing indexes were calculated using the ‘phenofit’ r package [64].

For the snow index, the time of snow melt in spring was defined as the initial occurrence of 50% snow cover, while the time of freezing in autumn was defined as the date when snow first attained 50% land cover [9]. Snow data was analyzed using MOD10A2 from 2015 to 2023, a MODIS snow cover product with 500-m spatial resolution and 8-day temporal resolution. The snow cover percentage in the buffer was calculated by overlaying the regional extent with MOD10A2 image maps and dividing the number of snow-covered raster cells in the overlapping region by the total number of raster cells. The resulting data were then linearly interpolated to derive daily snow cover [65].

Remote-sensing data to generate the above defined metrics were acquired from pixels within a 5-km radius buffer around specific locations the birds visited within two days after the start and before the end of the summer season [65] and each stopover site [22]. Areas not suitable for foraging, such as water, forest, woodland, urban, and built-up areas, within the buffer were excluded [66], based on the MODIS land cover product MCD12Q1 [67]. Stopover sites located south of the average zero-degree isotherm line in January (i.e. Qinling-Huai River line; Fig. 1d and e) were removed, due to the lack of zero-degree temperature and long-term snow under the warm climate.

### Analyzing the difference in the date of migration

All the analyses were conducted within r [68].

We utilized generalized linear mixed models (GLMMs) to examine the variability in the start and end date of migration. The observed migration dates of individual tagged geese using the methods described above were used as the response variable in the models, with four geographical groups and two species as explanatory variables and using year and individual as random variables to account for non-independence in date within years and individuals. The GLMMs were fitted with a Gaussian distribution and identity link using the ‘lme4’ r package. For post hoc comparisons of the GLMMs in group and species terms, we used the ‘emmeans’ r package (version 1.10.3) and applied a separate Tukey adjustment to the *p*-values [69].

### Analyzing whether geese are “surfers” of environmental metrics

We utilized data from all the migration nodes (including spring arrival and autumn departure nodes in breeding grounds and stopover sites) to conduct mixed-effect linear models (LMMs) using the ‘lme4’ r package. We utilized maximum likelihood estimates per group per season to conduct Simple Conventional Correlation tests of migration and environmental metrics. The observed arrival date in spring and departure date in autumn (Dobs) served as the response variables. Predicted arrival and departure dates described above (Dpre, i.e. nine environmental metrics) were the explanatory variables on a case-by-case basis, with individuals nested within year of migration as random variables on both intercept and slope. If all individuals within a group contained only single-year data, we only used individual as random variables on both intercept and slope [22].

Based on the expectation that goose migration perfectly matches the patterns of seasonal change in a particular metric (i.e. that they “surf” along the spring or autumn wave of that metric; see Fig. S4), we designated groups as “surfers” on a particular environmental metric when they satisfied two conditions: (1) a significant positive slope (*p* < 0.05) with 0 < lower 95% CI ≤ 1 and upper ≥ 1, and (2) a nonsignificant intercept (*p* > 0.05). Groups were designated as “weak surfers” if they met either of the following conditions: (1) a significant positive slope with lower 95% CI > 0 and upper < 1, or lower > 1 and any intercept, or (2) a significant positive slope and an intercept significantly different from zero [22, 70].

### Analyzing variation in arrival and departure probability

The probability of birds arriving in spring and departing in autumn was estimated using GLMMs and Maximum Likelihood Laplace Approximation. The probability of departure in autumn was defined as a value between 0 and 1, with 0 indicating staying at the breeding site or a specific stopover site and 1 indicating departure from that site. Therefore, the probability of departure at breeding or stopover sites for localities was set to 0, except for the last record before bird departure, which was set to 1 and the arrival probability in spring was defined using similar criteria [20]. We then calculated the number of days of a bird’s presence from the onset of environmental metrics (i.e., Dobs - Dpre). For example, a positive value of “Dobs - LSTa” indicates a bird arrived at a specific stopover site after the average daily LST reached 0℃, while a negative value indicates arrival before it. These new probability-related metrics were set as explanatory variables, and individual nested within year were included as random variables.

The numeric explanatory variables were standardized using z-transformation to ensure uniform variability. All variables in the model underwent collinearity testing using the ‘vif’ function of the ‘usdm’ r package, and only variables with a VIF less than 10 were retained [71]. To obtain the posterior distribution, 2,000 values were directly simulated from the joint posterior distribution of the model parameters using the ‘sim’ function of the ‘arm’ r package to obtain 95% CI of coefficients [72]. The effect of a predictor was considered significant when the corresponding 95% CI did not include zero [73]. By holding other variables at their mean value and manipulating only the variable of interest, we generated the response curve of the variables against the arrival and departure probability using the ‘predictorEffect’ function of the ‘effects’ r package [74–76].

## Results

### Difference of migratory departure and arrival date

**Fig. 2:**
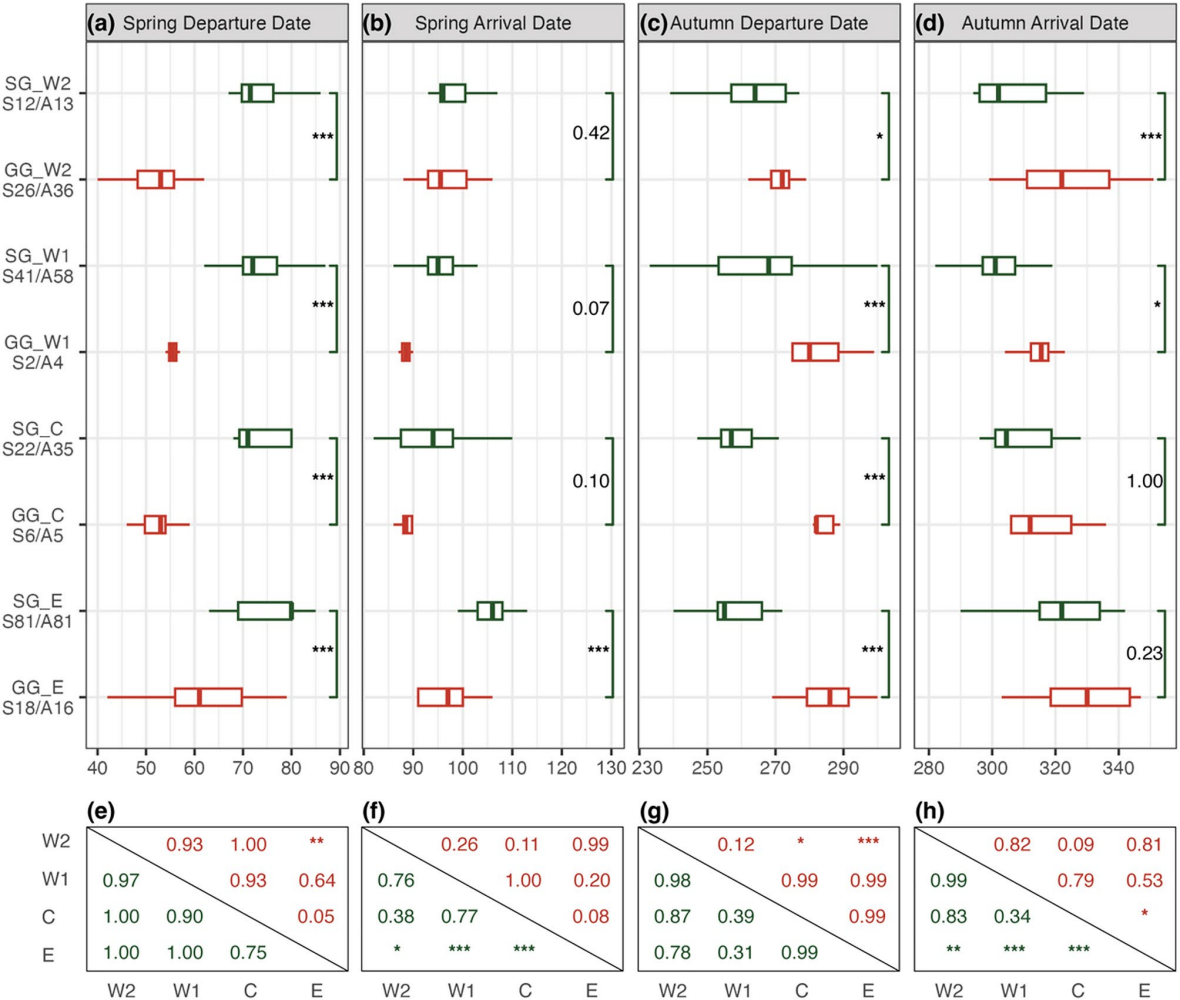
Comparison of migratory metrics between and within the two tagged geese species. **a**-**d**, boxplots illustrating differences between species; **e**-**h**, the *p*-value of differences between groups calculated by Tukey tests for post hoc analysis of generalized linear mixed models. Two colors indicate the Swan Goose (SG; green) and Greylag Goose (GG; red) respectively. Four shapes indicate the four different groups (W2, Western 2 group; W1, Western 1 group; C, Central group; E, Eastern group). In the convention ‘S*/A*’ the value for * indicates the number of migratory records in spring and autumn respectively. *p < 0.05, **p < 0.01 and ***p < 0.001

The tracked Swan Geese started their spring migration in mid-March and ended in early to mid-April (Table 1; average migration duration 28 ± 7 days). During this period, they travelled through the upper Yellow River (except the SG_E group), the mouth of Yalu River and the wetlands in Northeast China (only SG_E; see Fig. 1d). After breeding and moulting on the breeding grounds, they started autumn migration from mid to late September and finished between mid-October and late November (53 ± 19 days). In contrast to spring, they showed an increased preference for the Ordos Plateau (Fig. 1e). While all four groups started their migrations without significant differences in both seasons, the SG_E group ended their migration significantly later (Fig. 2f and g; *p* < 0.05). After excluding three autumn stopover sites situated south of the zero-degree isotherm in January, 470 spring arrival nodes (including 154 breeding and 316 stopover sites) and 580 autumn departure nodes (including 187 breeding and 393 stopover sites) were used for further analyses.

The tracked Greylag Geese started their spring migration between mid-February and mid-March and ended between late March and mid-April (Tables 1 and 40 ± 12 days). During this time, they staged in the middle and upper Yellow River (except GG_E) and the wetlands in Bohai Bay and Northeast China (only GG_E; Fig. 1d). Their autumn migration began between late September and late October and finished between early November and mid-December (48 ± 17 days). Among the four groups, GG_E started migration significantly later in spring and GG_W2 earlier in autumn (Fig. 2e and g; *p* < 0.01), while all groups ended without significant differences. After excluding two spring and four autumn stopover sites located south of the zero-degree isotherm, 206 spring arrival nodes (52 and 154) and 206 autumn departure nodes (61 and 145) were used for further analyses.

Unlike SG, GG (except GG_E) used the wetlands of Heyang Huanghe (35.15° N, 110.33° E) and Weinan Sanhe (34.97° N, 110.27° E) as stopover sites, but did not stop at the Ordos (Fig. 1d and e). These two wetland complexes are Important Bird and Biodiversity Areas, situated between the Hukou Falls and Sanmenxia Dam, close to the average zero-degree isotherm line in January. Additionally, GG_E did not stop at the mouth of the Yalu River but instead chose wetlands in the southern part of Bohai Bay. Swan Geese generally started spring migration later than Greylag Geese (Fig. 2a; *p* < 0.001) but ended on a similar date, except for the Eastern group, where SG_E ended significantly later than GG_E (Fig. 2b; *p* < 0.001). Moreover, Swan Geese typically started autumn migration earlier than Greylag Geese (Fig. 2c; *p* < 0.05) and ended earlier in the two Western groups (Fig. 2d; *p* < 0.05).

**Table 1:** The migratory metrics of tracked Swan Goose *Anser cygnoides* and Greylag Goose *A. anser*. Migratory metrics included average departure and arrival dates, total number of stopover sites, the number of migratory records (N) and tracked individuals (n)

Season	Group	Swan Goose				Greylag Goose			
		Departure date	Arrival date	Stopover (n)	N/n	Departure date	Arrival date	Stopover (n)	N/n
Spring	Western 2	Mar. 15 ± 5.8	Apr. 08 ± 4.6	25	12^a^/5	Feb. 22 ± 6.3	Apr. 06 ± 5.4	107	26/12
	Western 1	Mar. 14 ± 6.4	Apr. 06 ± 7.5	86	41/18	Feb. 25 ± 2.1	Mar. 29 ± 2.1	5	2/2
	Central	Mar. 15 ± 5.1	Apr. 04 ± 8.1	39	22/14	Feb. 21 ± 4.5	Mar. 30 ± 2.4	14	6/2
	Eastern	Mar. 17 ± 5.8	Apr. 16 ± 4.1	166	81^a^/81	Mar. 03 ± 10.9	Apr. 07 ± 5.8	30	18/8
	ALL	Mar. 16 ± 6	Apr. 11 ± 7.8	316	156/118	Feb. 25 ± 9	Apr. 05 ± 5.8	156	52/24
Autumn	Western 2	Sep. 20 ± 11.2	Nov. 04 ± 12.8	44	13/4	Sep. 28 ± 5.1	Nov. 19 ± 16.6	110	36^a^/15
	Western 1	Sep. 22 ± 14.5	Oct. 30 ± 11.2	130	58^b^/20	Oct. 11 ± 11.4	Nov. 10 ± 7.9	9	4/3
	Central	Sep. 17 ± 10.3	Nov. 05 ± 10.7	68	35^c^/21	Oct. 11 ± 3.6	Nov. 13 ± 13.2	8	5/3
	Eastern	Sep. 15 ± 7.3	Nov. 18 ± 12.2	154	81/81	Oct. 12 ± 9.7	Nov. 26 ± 14.5	22	16^a^/8
	ALL	Sep. 18 ± 11.2	Nov. 09 ± 14.4	396	187/126	Oct. 03 ± 9.6	Nov. 20 ± 15.7	149	61/29

Note: ^a^ one bird, ^b^ two birds, ^c^ five birds did not complete the migration;

### Surfers of environmental metrics

During the spring migration, almost all SG and GG groups showed weak surfing of four temperature metrics, but no relationship with two plant metrics (Table 2). Only half SG groups and one GG group showed weak surfing with Snow (Table 2). Among the relationships with LSTa, Ta and Tcum, most slopes were less than one, and the data points primarily clustered within the upper-left part of the panel, up the expected 1:1 relationship (see Fig. S5), indicating that the tracked birds migrated faster than the change rate in the metrics, but still arrived later at the breeding sites.

During the autumn migration, SG in the Western 2 and Eastern groups and GG in the Central and Eastern groups were almost weak surfers or surfers of the four temperature metrics (Table 2). SG in the Western 1 group and GG in the Western 2 group were surfers of Tcum. Only SG and GG in the East were weak surfers of Dormancy and half SG groups were weak surfers of Snow (Table 2). For the four temperature indexes, almost all SG groups started migration before them but moved at a slower pace (Fig. S6b; all slopes > 1), while GG groups migrated at a faster or similar rate with them (Fig. S6d; slopes < 1).

**Table 2:** The match between the arrival/departure date and the predicted date of environmental metrics in spring/autumn respectively. The level of support (from high to low) is marked in the left top of maps, as  for a surfer,  for a weak surfer, and  for a non-surfer (see Methods for explanation and definition)

Season	Group	LSTa	LSTn	Ta	Tcum	SOS/ EOS	Greenup/ Dormancy	Snow
Spring	SG_W2	◖	◖	◖	◖	○	○	◖
	SG_W1	◖	◖	◖	◖	○	○	○
	SG_C	◖	○	◖	◖	○	○	○
	SG_E	◖	◖	◖	◖	○	○	◖
	SG_ALL	○	◖	◖	◖	○	○	○
	GG_W2	◖	◖	◖	◖	○	◖	◖
	GG_W1	○	◖	○	○	○	○	○
	GG_C	●	◖	○	◖	○	○	○
	GG_E	◖	◖	◖	◖	○	○	○
	GG_ALL	◖	◖	◖	◖	○	○	◖
Autumn	SG_W2	◖	◖	●	◖	○	○	◖
	SG_W1	○	○	○	●	○	○	○
	SG_C	○	○	○	○	○	○	○
	SG_E	◖	◖	◖	◖	○	◖	◖
	SG_ALL	○	○	◖	○	○	○	◖
	GG_W2	◖	○	○	●	○	○	○
	GG_W1	○	◖	○	○	○	○	○
	GG_C	●	◖	◖	○	○	○	○
	GG_E	●	●	●	●	○	◖	○
	GG_ALL	○	○	○	○	◖	◖	◖

### Probability with environmental conditions

**Fig. 3:**
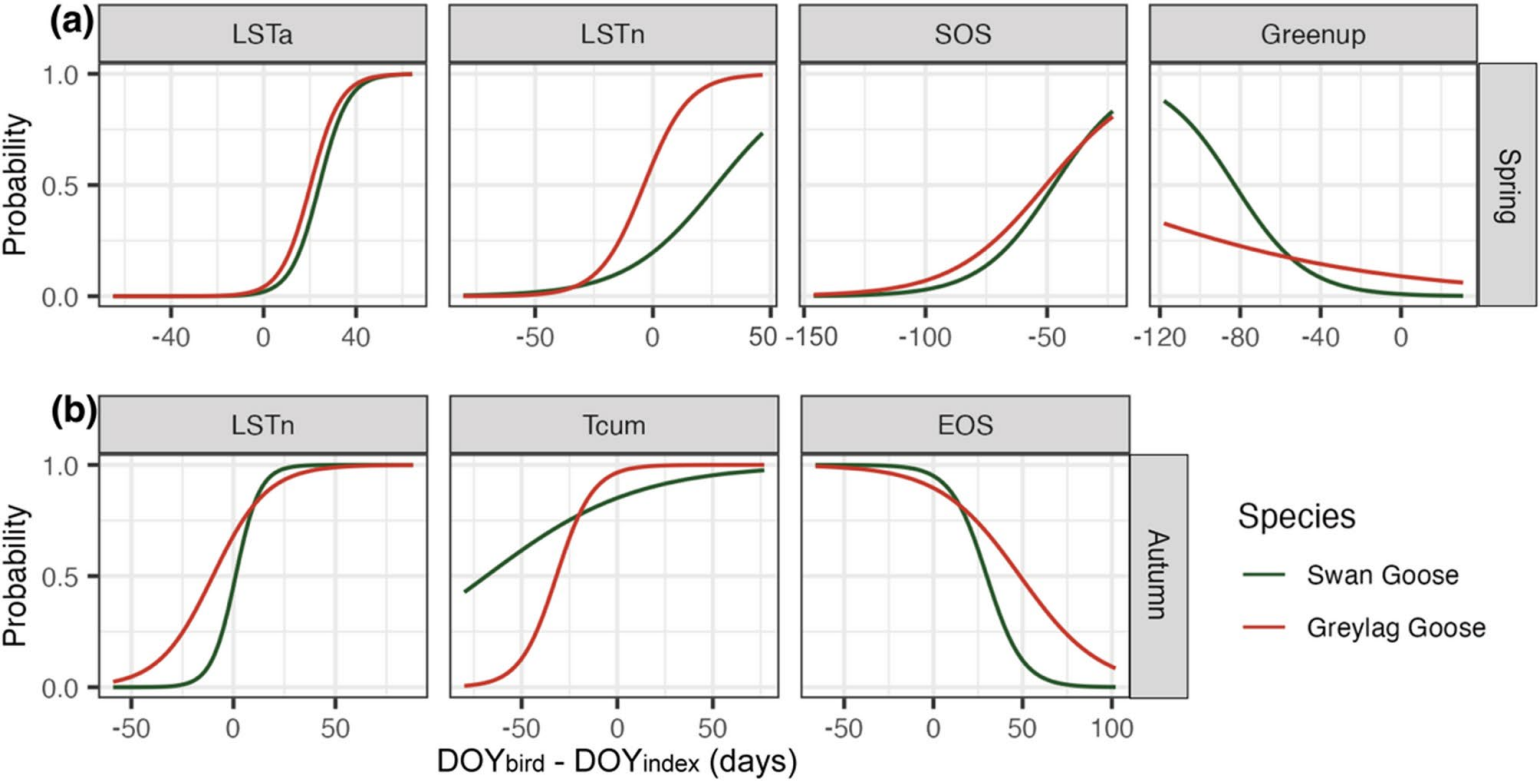
The effect of environmental conditions on the (**a**) spring arrival and (**b**) autumn departure probability at species level

The best-fit model for estimating the arriving probability in spring migration nodes included four variables: LSTa, LSTn, SOS and Greenup (Table 3 & S2). Both species showed similar responses to the change in LSTa, with an increasing arriving probability observed in the following month after LSTa (Fig. 3a). Except for W1 groups of the two species, there was a significant increase in arrival probability before LSTn (Fig. S7a). The probability increased as the date before the onset of Greenup and approached the SOS for two species.

For autumn migration, the best-fit model selected three variables: LSTn, Tcum and EOS (Table 3 & S2). The departure probability for the two species showed similar responses but different reaction rates to the change in the environment (Fig. 3b). The probability of SG was strongly associated with LSTn and EOS, and they preferred to depart after the onset of LSTn and before EOS. For Greylag Goose, the birds were more moderate in their response to the above two factors but preferred to depart close to the onset of Tcum.

Snow index was never selected in the model selection process, probably due to the fact that *c.* 28% of sites were not covered by snow. Other factors, such as the daily average air temperature (Ta), had high multicollinearity with the current fixed factors (Fig. S8), so they were eliminated from model selection.

**Table 3:** Key predictors included in the best-fit model for estimating the migration probability of two species. *p < 0.05, **p < 0.01 and ***p < 0.001

Type	Model	Conditional *R*^2^	Marginal *R*^2^	Fixed effect	Estimate	95% CI	*p*-value
Spring arrival probability	LSTa * Species + LSTn * Species + SOS * Species + Greenup * Species	0.86	0.76	LSTa	2.67	2.50–2.83	***
				LSTn	1.47	1.33–1.61	***
				SOS	1.18	1.03–1.32	***
				Greenup	-0.83	-0.98 - -0.68	***
Autumn departure probability	LSTn * Species + Tcum * Species + EOS * Species	0.80	0.68	LSTn	2.88	2.74–3.02	***
				Tcum	1.47	1.31–1.62	***
				EOS	-1.61	-1.70 - -1.52	***

## Discussion

Using a large dataset of spring and autumn migrations of Swan Goose and Greylag Goose from four breeding groups on the Mongolian Plateau, and a broad set of environmental metrics, we analyzed the difference in their migratory time, how they followed these metrics as indicators of environmental change, and the probability of spring arrival and autumn departure. We found: (1) There were significant differences in departure times between species in both seasons, yet their arrival times were similar. (2) Migration phenology of Eastern groups was significantly different from the Western and Central groups for both species. (3) The timing of migration in all groups from two species followed the four temperature indexes (LSTa, LSTn, Ta and Tcum) during spring and autumn migration seasons. Some of the groups also followed snow cover change, but generally did not respond to the vegetation indexes (SOS/Greenup in spring, EOS/Dormancy in autumn). (4) In spring, LSTa proved an effective predictor of arrival probability for both species. In autumn, SG exhibited a stronger response to LSTn, while GG displayed greater sensitivity to Tcum.

### Different migratory phenology

During spring, the departure time from the wintering grounds was significantly different between tracked groups (GG_W2/W1/C < GG_E < SG). This may be associated with the first stopover site used by geese. Listed by latitude, these were Heyang Huanghe and Weinan Sanhe Wetlands in the middle of Yellow River (35°N; for GG_W2/W1/C), the southern part of Bohai Bay (38°N; for GG_E), the mouth of the Yalu River (40°N; for SG_E), and the upper Yellow River (40.5°N; for SG_W2/W1/C). This sequential order aligns with the start time of spring migration. The reason why SG avoided using the wetlands in the middle of Yellow River is potentially due to lack of appropriate food and possibly interspecific competition (see below). The wetland types in this region primarily consist of reedbeds and mudflats [77], which are more compatible with the feeding habits of GG [78–80]. In contrast, SG mainly grub in soft open muddy substrate for the tubers of submerged plants or graze on graminoids like *Carex* spp [81]., restricting foraging in these areas. Moreover, this region is an important wintering site for Whooper Swan *Cygnus cygnus*, whose food also obtained by grubbing partly overlaps with Swan Goose [82], leading to potential interspecific competition. Towards the end of the spring migration, SG/GG_E arrived later than the others possibly due to lower temperatures in the eastern part of the Mongolian Plateau compared to elsewhere (Fig. S9a) [83]. SG_E arrived later than GG_E due to the higher breeding latitude of the former (*c.* 2°; Fig. 1b).

In autumn, SG started their migration earlier than GG, which aligns with field observations at Dalai Lake, China (117°N, 48°N) [84]. Both field observations and nest information calculated from tracking data (Table S1) showed similar laying and hatching times for both species, and a longer time required for the juvenile SG to fledge [85–87]. Therefore, the later start to migration in GG is not associated with the nest duration and juvenile growth, but rather potentially with the low rate of fat storage, perhaps related to the avoidance of competition [88]. Studies on wintering areas have shown that GG avoids competition with SG by staggering their foraging times [89, 90]. Hence, we predict that GG may forage on lower-quality food to avoid competition, thereby using a longer time to meet their energy requirements for moulting and migration [91], resulting in delayed start dates. Of course, it is also possible that, due to the limitation of the longer bill (SG, *c.*90 mm; GG, *c.*60 mm) [92] and contrasting gut microbiota [93], food suitable for SG was depleted earlier, forcing them to start autumn migration. These speculations need more field observations and tracking data analysis from breeding sites to be verified. GG_W2 seemed to initiate migration earlier than the other three GG Groups, possibly due to the lower nighttime temperatures (Fig. S9b).

The different migration strategies between SG and GG may partially explain their opposing population trends. During spring migration, GG arrived at breeding grounds earlier, enabling them to secure optimal nesting sites and enhance reproductive fitness [94], thereby improving first-year survival rates during initial migration [95]. In contrast, SG initiated autumn migration earlier with longer migration duration, increasing their exposure to additional mortality, including hunting pressure, particularly among the juveniles [96, 97]. For the eastern group, SG exhibited longer migration distance and demonstrated high dependence on the Yalu River Estuary as an important stopover site (Fig. 1d and e), which not only elevated migratory risks but also rendered them vulnerable to declining habitat quality in this critical wetland [98]. Another key factor is their dietary divergence. On the wintering grounds, SG primarily foraged on buried tubers of *Vallisneria spp.*, a food resource that has become increasingly scarce due to hydrological changes, aquaculture expansion, and pollution [81]. In contrast, GG have progressively shifted to agricultural fields during the non-breeding season [78], achieving higher energy and nutrient intake rates compared to natural habitats [99]. These factors collectively contributed to the significantly lower age ratio observed in SG relative to GG (mean 0.128 vs. 0.188 during 2016–2019 [100]),, ultimately driving their opposing population trends.

### Similar reproductive phenology

Through the analysis of tracked female adults, we obtained 80 breeding records (Table S1), indicating that the juveniles of two species could match with food growth peak. Despite arriving at the breeding site before vegetation green-up (i.e. overtaking Greenup), both species finished hatching around the 140th − 150th day of the year, coinciding with the time of peak vegetation growth (SOS in Fig. S8), enabling the juveniles to rapidly gain enough energy to successfully fledge and accumulate sufficient fat for migration before the food quality declines [101].

Our findings underline the largely “income” breeding strategy adopted by SG and GG: geese, which spent three weeks on pre-incubation feeding activities in the breeding grounds (Table S1). Compared to Arctic-breeding geese, the spring migration distance of SG and GG is *c.*60% of other species and only *c.*30% of their migration duration [102], although with a longer pre-incubation period on the breeding grounds [9, 103]. This indicated SG and GG may shift the intake of fat and nutrient stores for reproduction from the spring stopover sites to close to, or at the breeding grounds, so the food conditions on the breeding grounds would more greatly influence their clutch size [104]. The reason why our two species did not forage for extended periods at stopover sites may be associated with the Greenup date in these sites, which occurs later than on the breeding grounds (Figs. S8a & S8c). Therefore, unlike the importance of food support by stopover sites for Arctic-breeding geese [105, 106], the attraction of the food availability on the breeding grounds (and potentially even wintering grounds) relative to the staging grounds may play a more important role in the reproduction and survival of these two species.

### Comparison of environmental predictors

For Arctic-breeding geese, the green wave and snow cover are commonly used as environmental predictors of their migration process. For instance, both the Barnacle Goose *Branta leucopsis* and the Greater White-fronted Goose *A. albifrons* in Europe adjusted their spring migration to track peak vegetation growth [22]. Moreover, Barnacle Geese have been observed to accelerate their spring migration in response to earlier snowmelt [9]. However, our findings suggest that these two indices are not suitable for predicting the migration process of SG and GG breeding on the Mongolian Plateau.

Our results indicate that both SG and GG do not follow the vegetation indexes during their migrations, including SOS and Greenup in spring, and EOS and Dormancy in autumn. Kelly et al.(2016; [107]) attributed the lack of support for the green wave hypothesis to the lack of seasonal south-to-north greening derived from remotely sensed indices estimated. Indeed, we found the vegetation following migratory routes of the geese did not perform the expected gradation of greening or yellowing with latitude change, but rather exhibiting a near-synchronous growth trend from the Mongolian Plateau to the Yangtze River floodplain (Fig. S8). Even the vegetation at the stopovers located along the upper Yellow River started growth later and ended earlier than that in the breeding sites [108, 109]. This results in the EOS being a good predictor of geese leaving the breeding site (in Fig. 3b, geese departed after LSTn and before EOS; here, EOS occurred after LSTn), but it becomes unreliable in the latter migratory period, due to the crossover with backwards-shifting temperature indexes (in these sites, EOS occurred before LSTn). Therefore, we think the above vegetation indexes are not appropriate indicators to predict the whole migration process of herbivorous birds within this range despite the obvious expectations. Jiang et al. (2019; [110]) proposed a method by defining the minimum NDVI in snow-covered areas as 0.08, in doing so, he was able to obtain a relationship between green-up date and latitude. Although this approach lacks a firm theoretical basis, this may offer an opportunity to enhance the use of vegetation indices in future studies.

Tracked geese gradually overtook the date of 50% snow cover in spring and remained ahead of it throughout autumn, which is consistent with the findings for other geese and swans [17, 23, 65]. However, the snow index calculated by the MODIS data revealed a null value of *c.* 28%, particularly in the stopover sites along the Yellow River and the Liao River in Northeast China (Fig. S10). Similar results are observed in the study based on passive microwave remote sensing records [111], confirming that these regions were without long-term snow cover, rather than a gap in the MODIS data. A recent study showed the continuous snow cover days in each snow year of these regions were less than 10 days from 1981 to 2019 [112]. Consequently, the snow index is a correlative, but not suitable parameter to predict the migration process of these birds. Since the snow index was closely related to the other three temperature indexes (LSTa, Ta, and Tcum; Fig. S8), the other three provide better alternatives.

During spring migration, both species followed behind LSTa, Ta and Tcum and gradually approached them at breeding sites. The arrival probability rapidly increased after the land surface temperatures exceeded 0℃ (LSTa is the most representative, due to high covariance among them). During this phase, the soil begins to thaw continuously [113], making the substrate soft, allowing moisture to enter the active layer, creating favourable temperature and moisture conditions for vegetation growth [114] and for geese to grub for subterranean plant storage organs. Therefore, geese could obtain part of the energy for migration and reproduction, by feeding on reed rhizomes and tubers [78, 79, 81].

In autumn, geese migrated ahead of the three indexes, when above ground green biomass production ceased, graminoid leaf quality declined and the soil froze, making it challenging for geese to access food from beneath the soil. Additionally, the autumn migration cues of the two species were different: SG respond more rapidly to LSTn, when soil begins to freeze at night, consistent with Xu and Si (2019; [20]), while GG showed a more rapid response to Tcum. Overall, using temperature parameters to predict the migration progress of both species appears to be more reasonable.

In this study, we focused on temperature, vegetation, and snow as climatic drivers of goose migration. However, it is important to recognize that migratory behavior is also influenced by a complex interplay of additional environmental factors, such as wind conditions [54], precipitation [20], snowfall [115] and freeze-thaw cycle [23]. Furthermore, migration timing is modulated by the remaining distance to the destination [73] and the progress of the migratory season [116]. While our current analysis does not comprehensively quantify these additional influences, we emphasize the need for future studies to develop more advanced process-based phenological models that can better integrate these multifaceted environmental drivers.

## Conclusions

In this study, we systematically analysed the migratory phenology and their likely environmental drivers among four geographically discrete groups of two goose species (Swan Goose and Greylag Goose) which bred across the Mongolian Plateau. We found the two goose species follow behind the 0 °C daily average temperature and before the green-up of plants on their spring migration, allowing the timing of their breeding seasons to time hatching of goslings to coincide with the plant growth peak, ensuring a steady food supply for the nidifugous juveniles. The nighttime and cumulative temperatures were important cues to predict the two species’ respective autumn migration timing. Due to later green-up and earlier dormancy of plants in stopover sites, the food availability on the summering grounds and potentially even on the wintering quarters are crucial for the reproduction of two species. Our findings could help quantify the effects of ongoing and rapid environmental change on bird migration and survival. We particularly recommend follow-up studies to monitor the effects of continuing climate change on food availability during their migration and on fitness measures to better quantify impacts on their population dynamics.

## Electronic supplementary material

Below is the link to the electronic supplementary material.

Supplementary Material 1

## Data Availability

No datasets were generated or analysed during the current study.
