# Supplementary material for: Exploring potential environmental drivers of migration phenology in two Mongolian Plateau-nesting goose species
Source: Mov Ecol. 2025 Aug 14;13:58. doi: 10.1186/s40462-025-00583-0 (PMC12351783; doi:10.1186/s40462-025-00583-0)
Supplement: Supplementary file 1 — Supplementary Material 1 [file 40462_2025_583_MOESM1_ESM.docx]

# **Appendix A. Supplementary data**


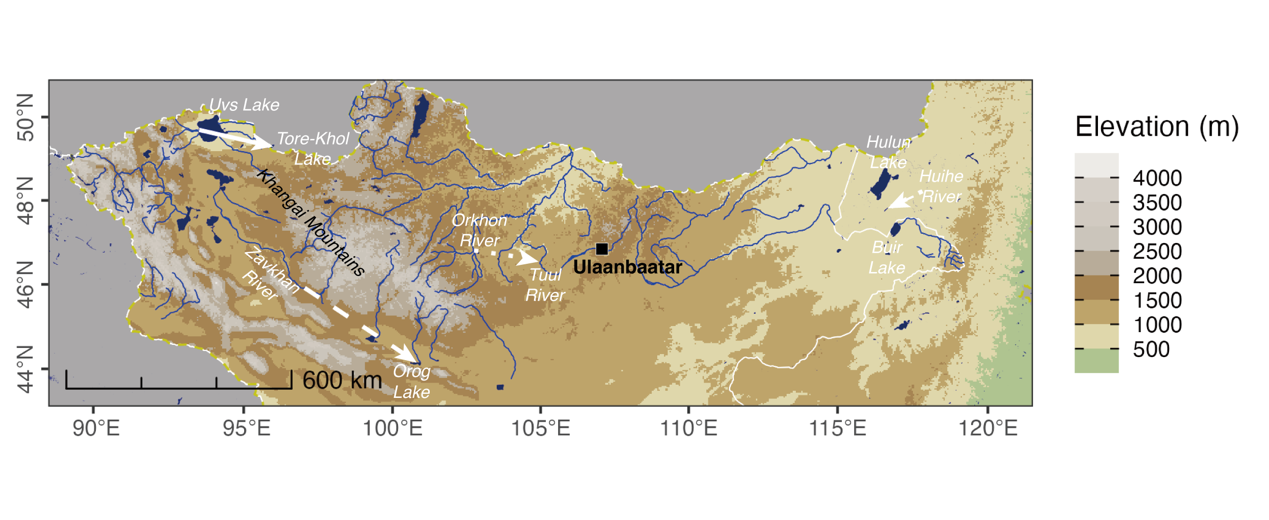


**Fig. S1.** The moult migrations (shows as white arrows) of tracked geese from the four defined geographical groups. Geese from Western 2 Group bred and moulted in the Uvs lakes, while some moulted in the wetlands around Tore-Khol Lake (50° N, 95° E). Western 1 Group bred and moulted along the Zavkhan River, while some flew *c.* 300 km to the Orog Lake (45.1° N, 100.8° E) to moult in late August. Central Groups bred along the Orkhon River, while almost all flew *c.*200 km to Tuul River to moult in August. Eastern Group bred and moulted in the Dauria Region, Huihe River and Xilinhot Grassland.


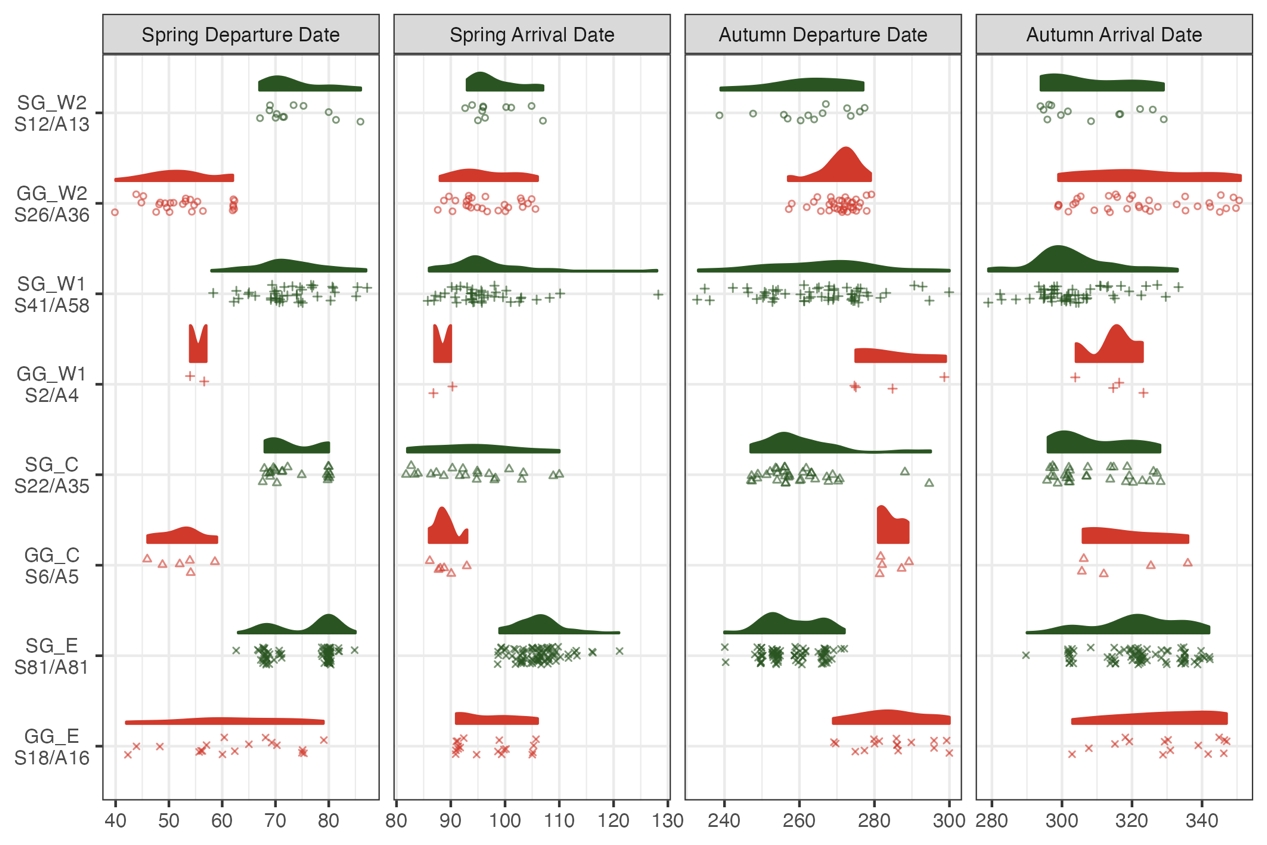


**Fig. S2.** Half-violin plots illustrating the distribution of migration timing. Two colours indicate the Swan Goose (SG; green) and Greylag Goose (GG; red) respectively. Four shapes indicate the four different groups (W2, Western 2 group; W1, Western 1 group; C, Central group; E, Eastern group). In the convention ‘S*/A*’ the value for * indicates the number of migratory records in spring and autumn respectively.


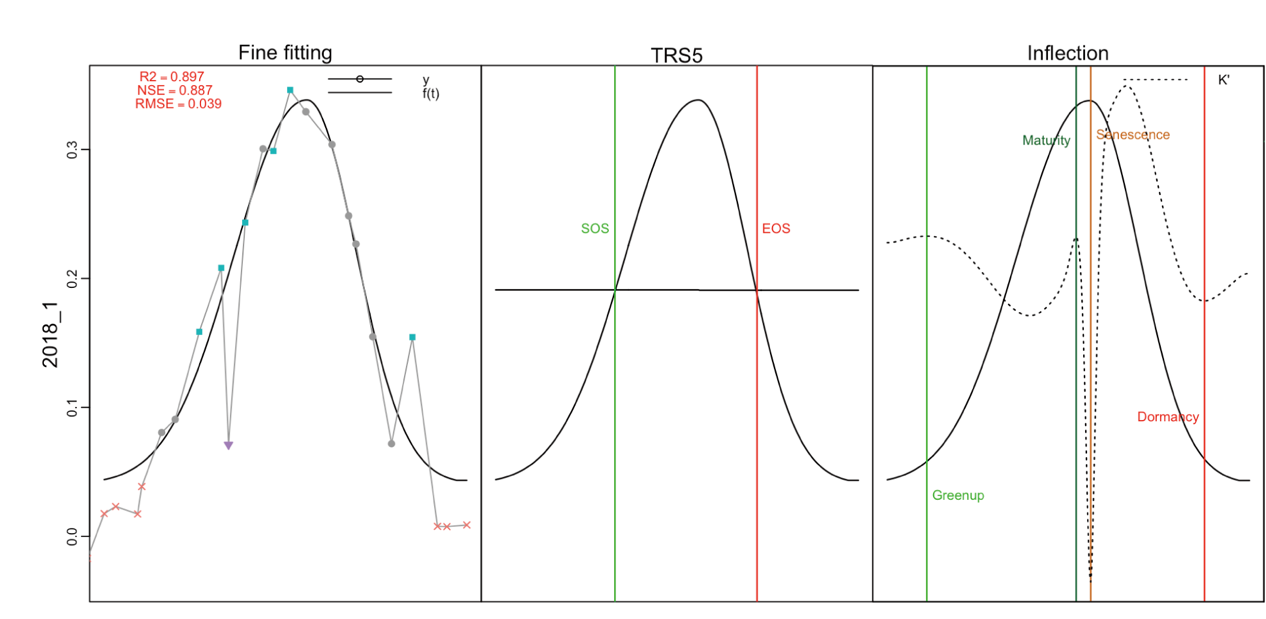


**Fig. S3.** An example of plant phenology extraction by Threshold and Inflection methods. (1) The left is the fitted NDVI time series by weighted Whittaker (*wWHIT*) rough fitting and Asymmetric Gaussian (AG) fine fitting. The crosses and inverted triangles are the NDVI values divided into snow and cloud in the MODIS QA file respectively, others are the data with high quantality. (2) The middle is the start and end date of the growing season (SOS & EOS), which are extracted by a 50% threshold. The Threshold method was based on the rescaled normalized vegetation time series (NDVI_ratio_) in the range of [0, 1] of each year, as is the threshold. The formula is NDVI_ratio_ = (NDVI_t_ – NDVI_min_)/ (NDVI_max_ – NDVI_min_). (3) The right is the date of Greenup and Dormancy extracted through the Inflection method, which is based on the change rate of curvature (K’, dashed line). Greenup was the first local maxima and Dormancy was the last local of the change rate [1].


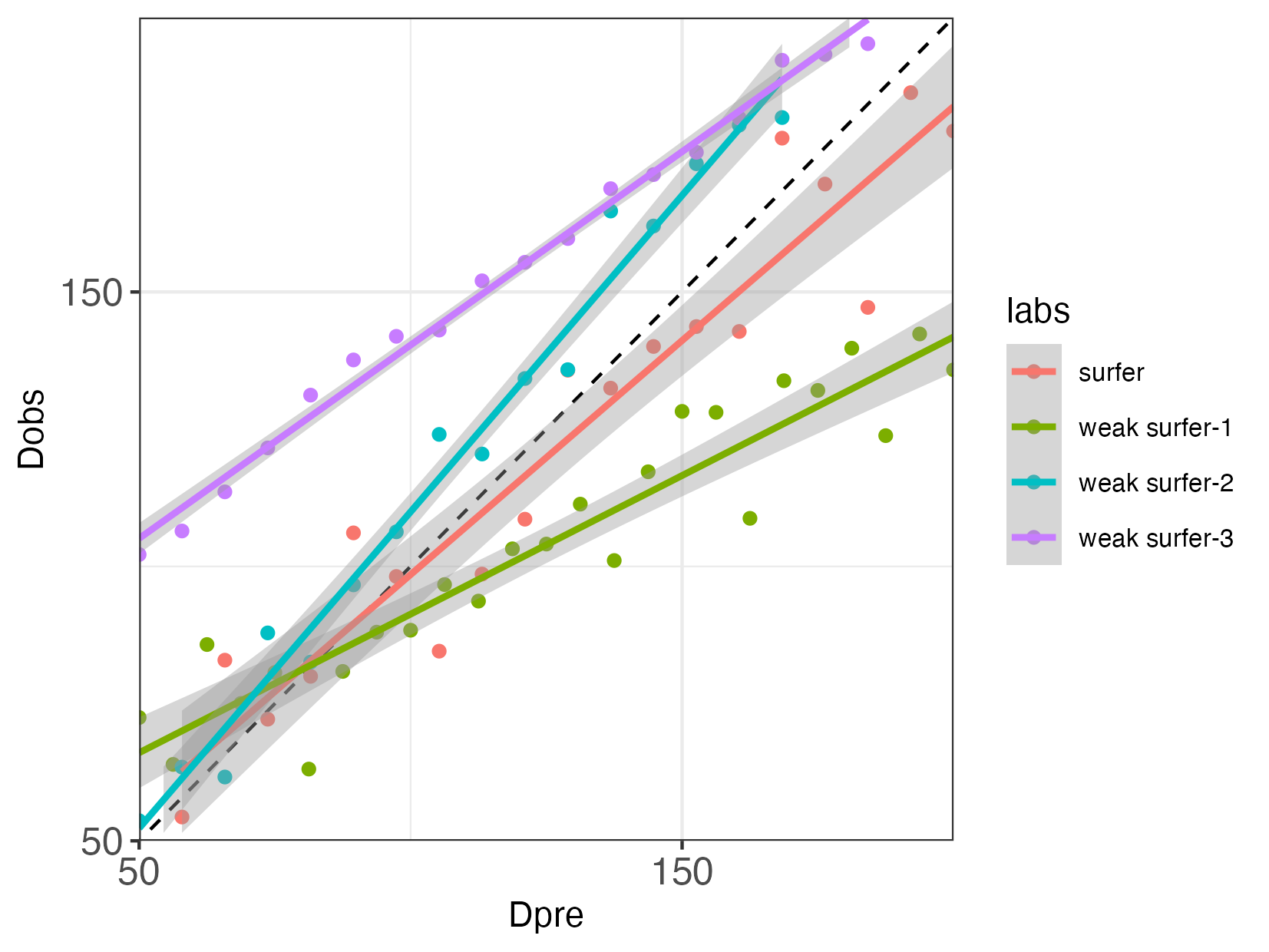


**Fig. S4.** The schematic plot describing surfer scenarios. A group is designated as “surfer” on a particular environmental metric when they satisfied two conditions: (1) a significant positive slope (*p* < 0.05) with 0 < lower 95% CI ≤ 1 and upper ≥1, and (2) a nonsignificant intercept (*p* > 0.05). Groups were designated as “weak surfers” if they met either of the following conditions: (1) a significant positive slope with lower 95% CI > 0 and upper < 1 (“weak surfer-1”), or lower >1 (“weak surfer-2”) and any intercept, or (2) a significant positive slope and an intercept significantly different from zero (“weak surfer-3”) [2,3].


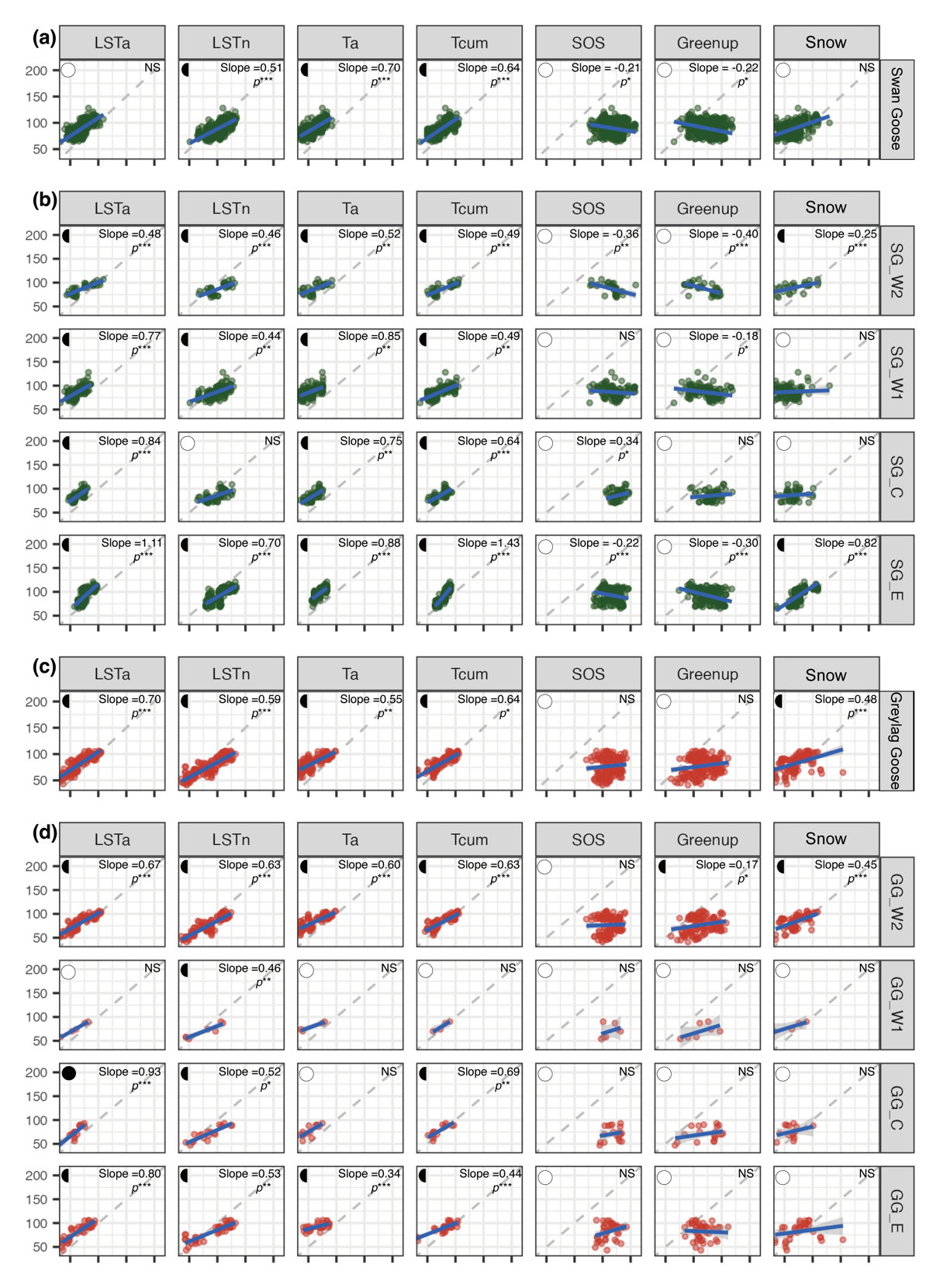


**Fig. S5.** The match between the arrival date and the predicted date of environmental metrics in spring. a & c, the Simple Conventional Correlation result in species level; b & d, group level. The x and y axes denote the predicted arrival day of the year at stopover and summering sites and the observed arrival day by birds, respectively. The grey dashed lines with slope = 1 and intercept = 0 indicate the trajectory of a perfect match between timing of migration and environmental metrics. Plotted points to the upper left of the lines indicates that the bird arrived after the expected dates, to the lower right, before. The level of support (from high to low) is marked in the left top of maps, as
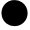
 for a surfer,
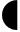
 for a weak surfer, and
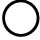
 for a non-surfer (see Methods for explanation and definition). The coefficient of the slopes and p-values are provided in the right top; otherwise, NS denotes an insignificant slope. **p* < 0.05, ***p* < 0.01 and ****p* < 0.001. Blue lines show the fitted result in models, and grey bands are the prediction intervals of the models. LSTa and LSTn are the date when the daily average and nighttime land surface temperature exceeded 0°C; Ta and Tcum are the date when daily average and 2-week cumulative air temperature exceeded 0°C; SOS and Greenup, represent the start date of plant growing season as estimated by the 50% threshold and Inflection method; Snow, the date when snow cover fell below 50%.

**
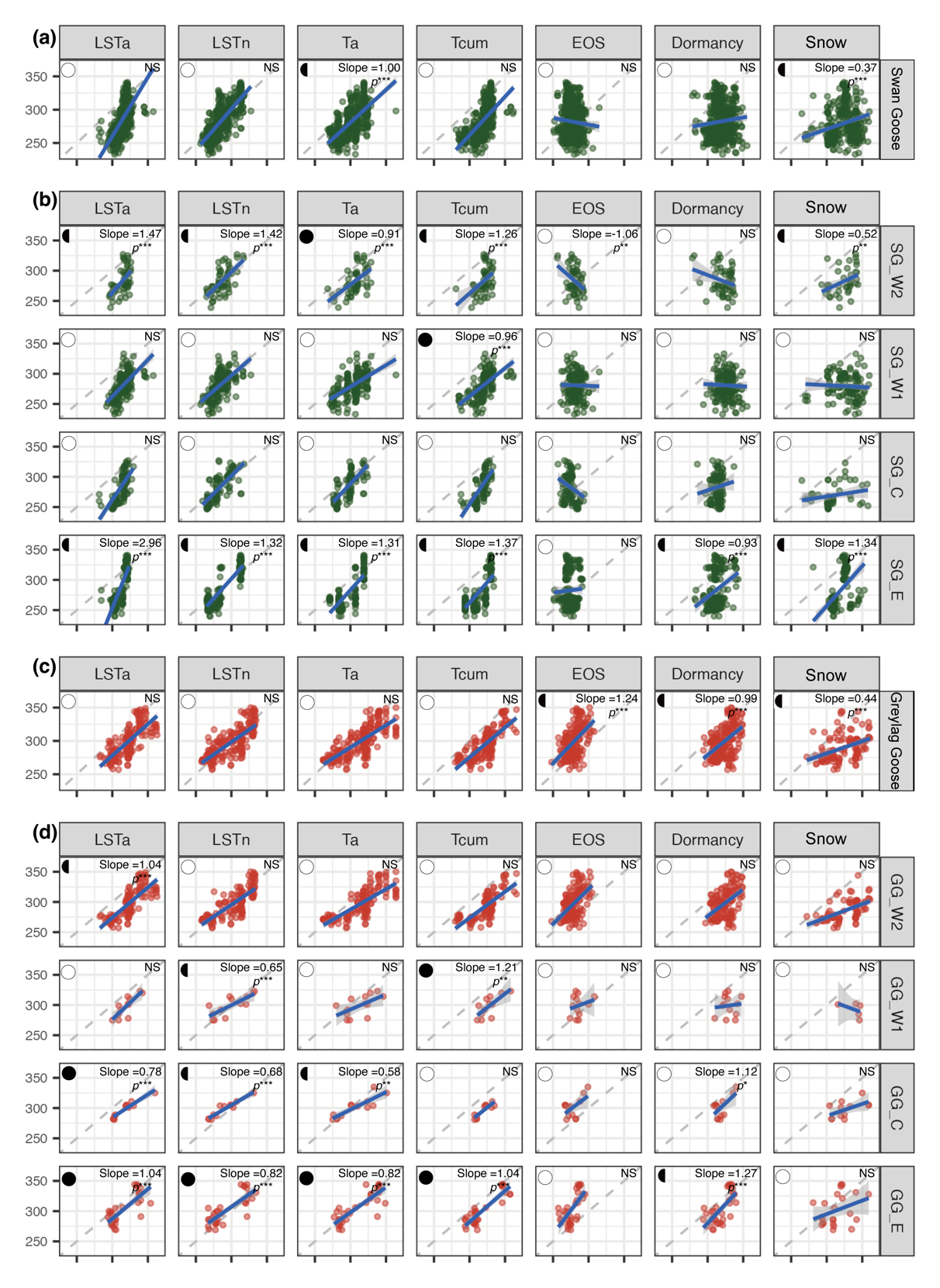
**

**Fig. S6.** The match between the departure date and the predicted date of environmental metrics in autumn. a & c, the Simple Conventional Correlation result in species level; b & d, group level. The x and y axes denote the predicted departure day of the year at summering and stopover sites and the observed departure day by birds, respectively. The grey dashed lines with slope = 1 and intercept = 0 indicate the trajectory of a perfect match between timing of migration and environmental metrics. Plotted points to the upper left of the lines indicate the bird departed after the expected dates, to the lower right, before. The level of support (from high to low) is marked at the left top of maps, as
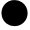
 for a surfer,
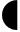
 for a weak surfer, and
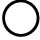
 for a non-surfer. The coefficient of the slope and p-value are provided at the right top; otherwise, NS denotes an insignificant slope. **p* < 0.05, ***p* < 0.01 and ****p* < 0.001. Blue lines show the fitted result in models, and grey bands are the prediction intervals of the models. LSTa and LSTn are the date when the daily average and nighttime land surface temperature fell below 0°C; Ta and Tcum, are date when daily average and 2-week cumulative air temperature fell below 0°C; EOS and Dormancy, represent the end date of plant growing season as estimated by the 50% threshold and Inflection method; Snow, the date when snow cover exceeded 50%.


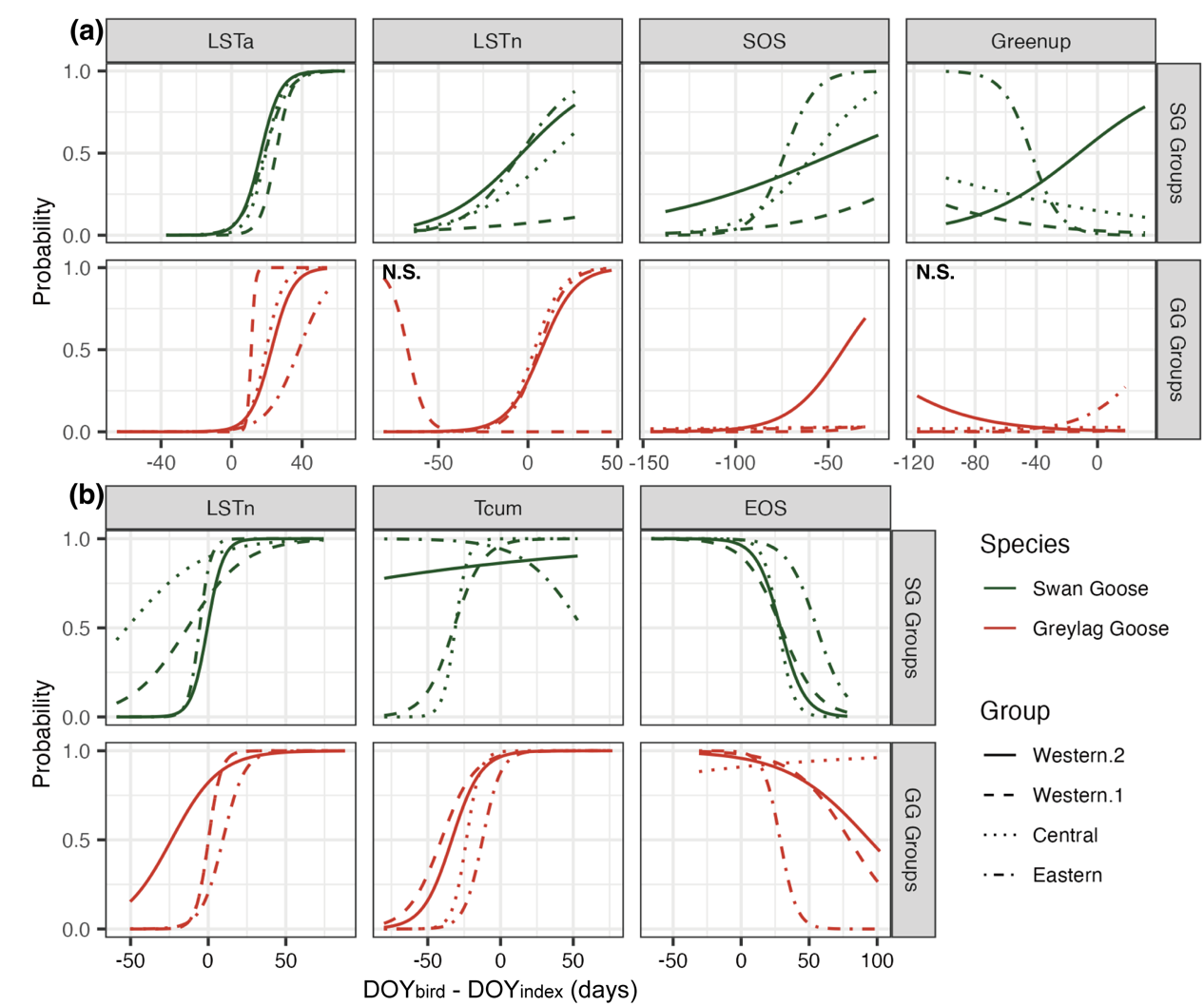


**Fig. S7.** The effect of environmental conditions on the (a) spring arrival and (b) autumn departure probability at group level. N.S. denotes no significant result.


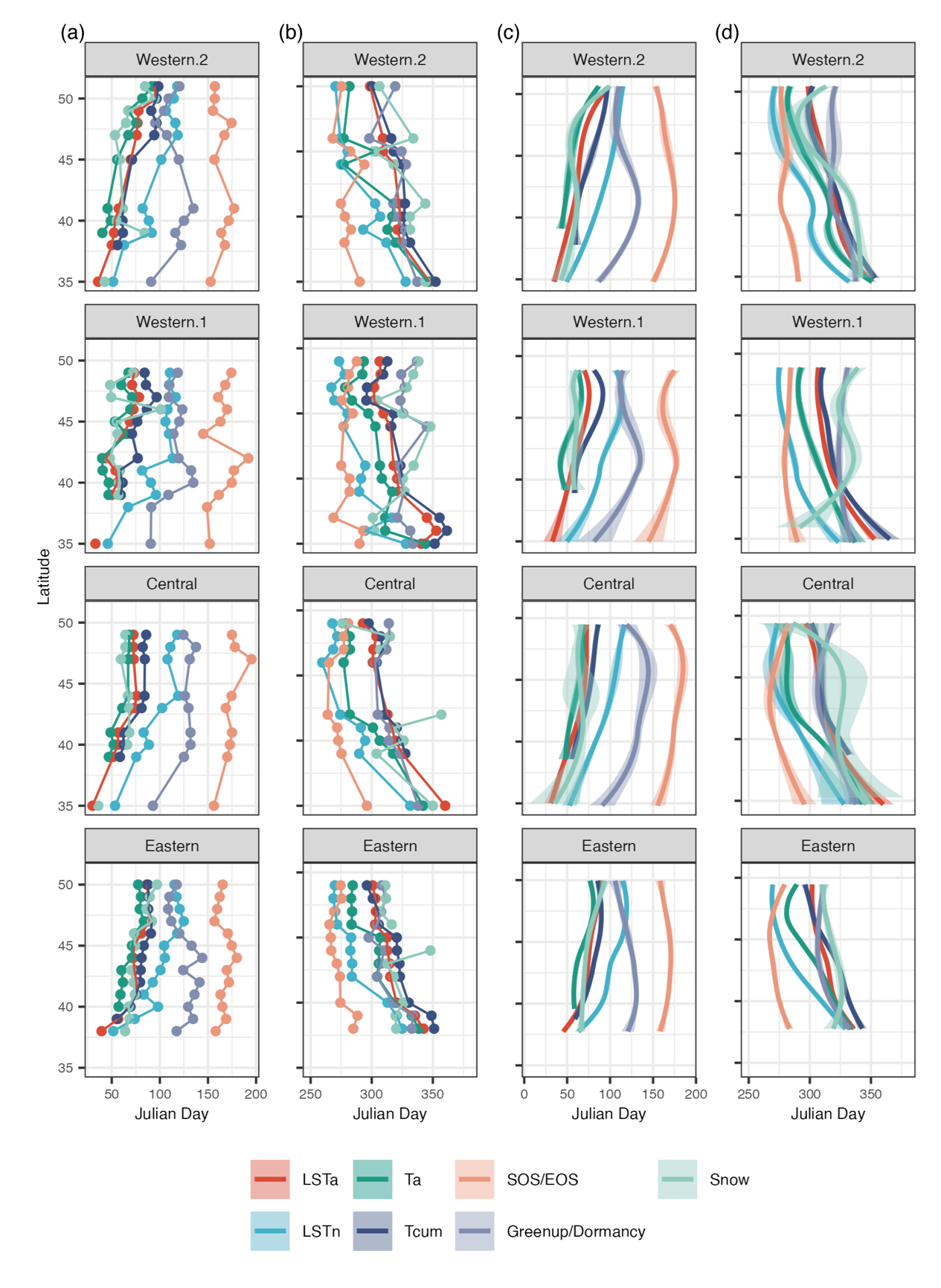


**Fig. S8.** Date of the nine environmental metrics at each migration node in spring (a & c) and autumn (b & d) for geese from four groups. The dots in a & b are the averages grouped by one latitude; lines in c & d are fitted results by the ‘loess’ method.


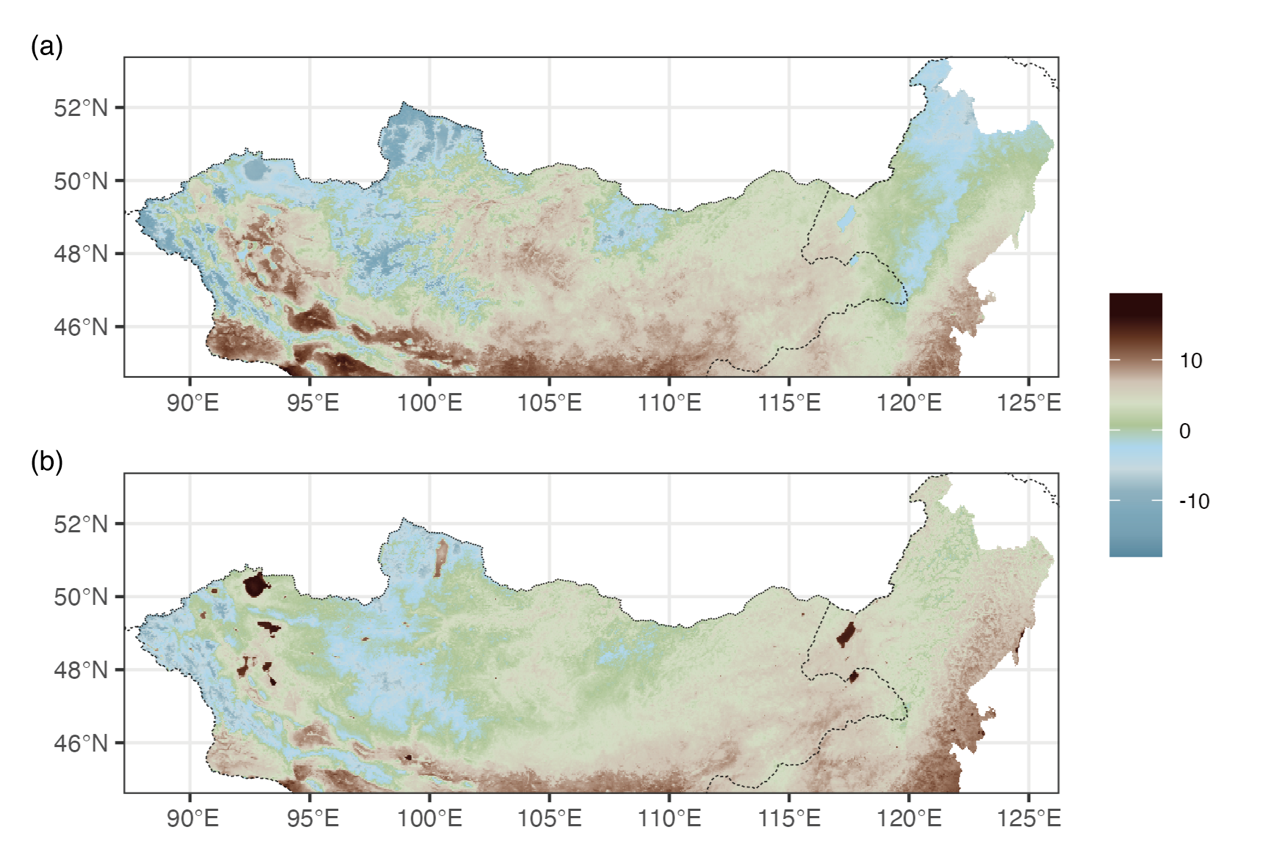


**Fig. S9.** The average land surface temperature on the Mongolian Plateau from 2015 to 2023. (a) the daily average land surface temperature of the 89^th^ day of the year, (b) nighttime of the 265^th^ day. DOY89 is the day close to the spring arrival date of the Western and Central Groups by tracked geese; DOY265 is the day close to the autumn departure date of the GG_W2 groups. The black dashed line is the boundary of countries. Temperature data is from the MOD11A2 product.


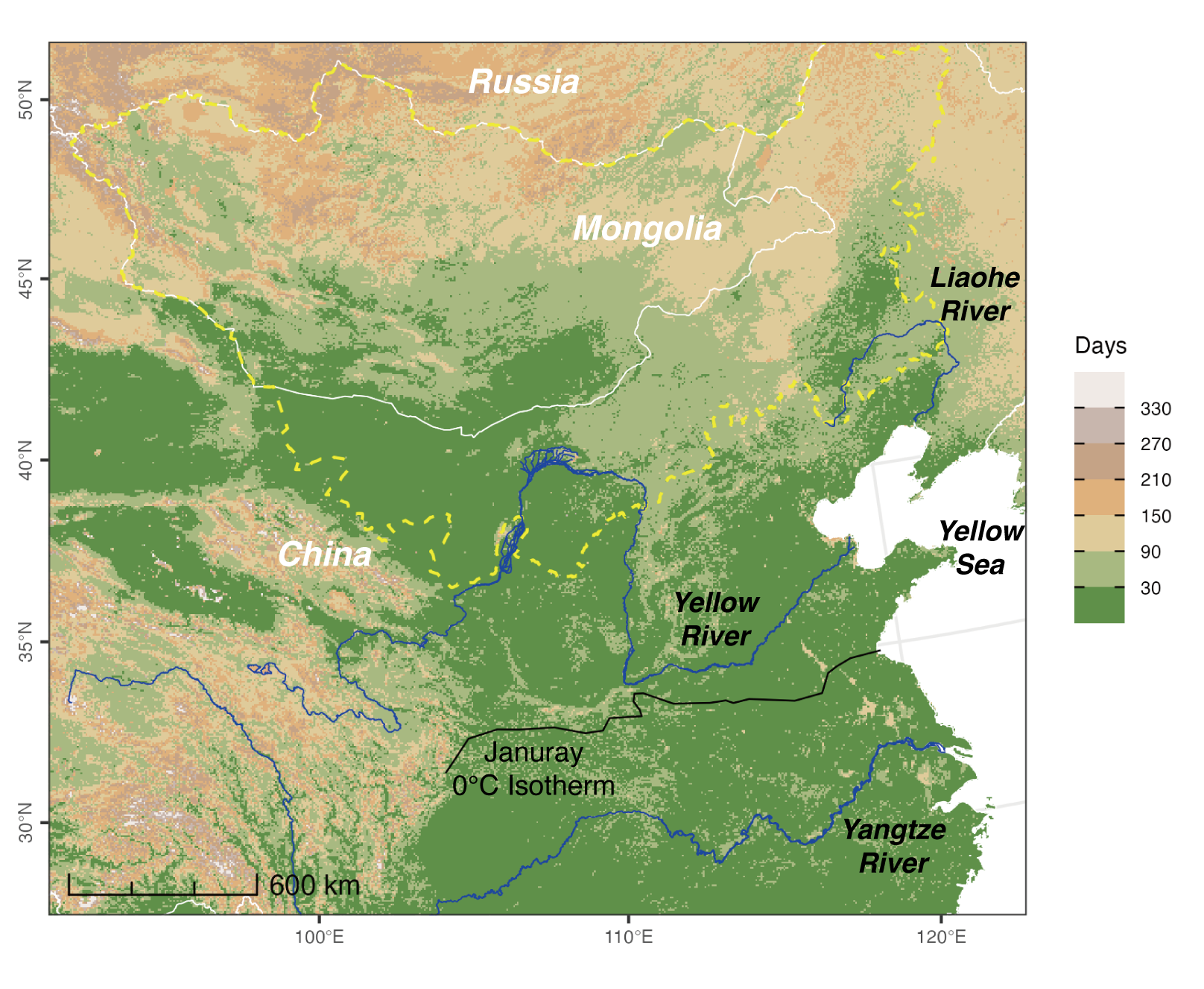


**Fig. S10.** Map of the average duration of annual snow cover during 2015 to 2023. Snow data is from the MOD10A2 product. The yellow dashed lines are the boundary of Mongolian Plateau, defined as the region consisting of Mongolia and the Inner Mongolia Autonomous Region of China; the white lines, boundary of countries; the blue lines, rivers; the black lines, the average zero-degree isotherm line in January.

**Table S1.** Summarized nesting information from 80 tracked female adult geese from 2018 to 2023. The start and end dates of the nest were estimated by the ‘nestR’ r package [4]. The nesting sites were determined as an area of < 200 m where at least 75% of the daily fixes were located for more than 10 days between late March and June [5,6]. The nest date was defined as the days when more than 75% of the fixes were at the nesting site. For the end and length of nesting, we excluded the individuals with a nest length < 21 days, which may have given up incubation due to disturbance.

| Species | Group | N | Spring arrival (Julian) | Pre- breeding (days) | Nest | | |
| --- | --- | --- | --- | --- | --- | --- | --- |
|  |  |  |  |  | Start (Julian) | End (Julian) | Length (days) |
| Swan Goose | Western 2 | 9 | 97 ± 4 | 26 ± 20 | 123 ± 19 | 151 ± 4 | 34 ± 2 |
|  | Western 1 | 23 | 97 ± 6 | 24 ± 14 | 121 ± 17 | 150 ± 14 | 30 ± 8 |
|  | Central | 7 | 90 ± 6 | 16 ± 9 | 106 ± 7 | 132 ± 3 | 23 ± 1 |
|  | Eastern | 27 | 105 ± 3 | 20 ± 15 | 125 ± 15 | 149 ± 15 | 27 ± 5 |
|  | ALL | 66 | 100 ± 7 | 22 ± 15 | 121 ± 16 | 148 ± 13 | 29 ± 7 |
| Greylag Goose | Western 2 | 7 | 95 ± 5 | 9 ± 4 | 104 ± 4 | 133 ± 8 | 30 ± 7 |
|  | Western 1 | 1 | 87 | 18 | 105 | 139 | 34 |
|  | Central | 4 | 90 ± 2 | 17 ± 5 | 107 ± 4 | 124 | 22 |
|  | Eastern | 2 | 92 ± 1 | 48 ± 25 | 140 ± 26 | 180 | 22 |
|  | ALL | 14 | 92 ± 4 | 18 ± 16 | 110 ± 15 | 138 ± 18 | 28 ± 6 |

**Table S2.** Key predictors included in the best-fit model for estimating the migration probability of two species at group level. **p* < 0.05, ***p* < 0.01 and ****p* < 0.001.

| Type | Groups | Model | Conditional R^2^ | Marginal R^2^ | Fixed effect | Estimate | 95% CI | *p*-value |
| --- | --- | --- | --- | --- | --- | --- | --- | --- |
| Spring arrival  probability | Swan Goose | LSTa * Groups +  LSTn * Groups +  SOS * Groups +  Greenup * Groups | 0.80 | 0.64 | LSTa | 2.51 | 2.31 - 2.72 | *** |
|  |  |  |  |  | LSTn | 0.64 | 0.49 - 0.80 | *** |
|  |  |  |  |  | SOS | 1.05 | 0.84 - 1.26 | *** |
|  |  |  |  |  | Greenup | -0.64 | -0.81 - -0.44 | *** |
|  | Greylag Goose | LSTa * Groups +  LSTn * Groups +  SOS * Groups +  Greenup * Groups | 0.91 | 0.85 | LSTa | 6.91 | 4.55 - 9.25 | *** |
|  |  |  |  |  | LSTn | 0.93 | -0.62 - 2.51 | 0.25 |
|  |  |  |  |  | SOS | 0.83 | 0.13 - 1.48 | * |
|  |  |  |  |  | Greenup | 0.50 | -0.14 - 1.21 | 0.15 |
| Autumn departure probability | Swan Goose | LSTn * Groups +  Tcum * Groups +  EOS * Groups | 0.86 | 0.77 | LSTn | 3.32 | 3.09 - 3.54 | *** |
|  |  |  |  |  | Tcum | 1.59 | 1.36 - 1.80 | *** |
|  |  |  |  |  | EOS | -2.55 | -2.69 - -2.43 | *** |
|  | Greylag Goose | LSTn * Groups +  Tcum * Groups +  EOS * Groups | 0.86 | 0.77 | LSTn | 4.37 | 3.47 - 5.26 | *** |
|  |  |  |  |  | Tcum | 3.27 | 2.47 - 4.14 | *** |
|  |  |  |  |  | EOS | -1.18 | -1.49 - -0.85 | *** |

# **References**

1. Zhang XY, Friedl MA, Schaaf CB, Strahler AH, Hodges JCF, Gao F, et al. Monitoring vegetation phenology using MODIS. Remote Sens Environ. 2003;84:471-5.

2. Wang X, Cao L, Fox AD, Fuller R, Griffin L, Mitchell C, et al. Stochastic simulations reveal few green wave surfing populations among spring migrating herbivorous waterfowl. Nat Commun. 2019;10:2187.

3. Aikens EO, Kauffman MJ, Merkle JA, Dwinnell SPH, Fralick GL, Monteith KL. The greenscape shapes surfing of resource waves in a large migratory herbivore. Ecol Lett. 2017;20:741-50.

4. Picardi S, Smith B, Boone M, Basille M. nestR: Estimation of Bird Nesting from Tracking Data. <https://github.com/picardis/nestR>. Accessed 15 May 2024.

5. Lameris TK, van der Jeugd HP, Eichhorn G, Dokter AM, Bouten W, Boom MP, et al. Arctic geese tune migration to a warming climate but still suffer from a phenological mismatch. Curr Biol. 2018;28:2467–73.

6. Zhu Q, Hobson KA, Zhao QS, Zhou YQ, Damba I, Batbayar N, et al. Migratory connectivity of Swan Geese based on species' distribution models, feather stable isotope assignment and satellite tracking. Divers Distrib. 2020;26:944-57.
